# Editorial for the Special Issue “Advances in Microbial Cell Factories”—Pioneering the Circular Bioeconomy

**DOI:** 10.3390/microorganisms14071578

**Published:** 2026-07-20

**Authors:** Thomas Brück, Dania Awad

**Affiliations:** 1 Werner Siemens Chair of Synthetic Biotechnology, Department of Chemistry, School of Natural Sciences, Technical University of Munich (TUM), D-85748 Garching bei München, Germany; 2 School of Engineering and Design, Department of Aerospace and Geodesy, Technical University of Munich (TUM), D-82024 Taufkirchen, Germany

The need to transition from a fossil resource-based economy to a sustainable, circular bioeconomy has never been more urgent. At the heart of this transition is the concept of the “cell-factory”, where microbial hosts are harnessed and programmed to convert various renewable feedstocks—such as agricultural waste, municipal biowaste, food processing side streams, and greenhouse gas emissions—into value-adding commodities. While traditional microbial fermentation has long been used for food products, antibiotics, and foundational therapeutics, recent leaps in systems biology, metabolic engineering, and bioprocess design have radically expanded the microbial product profile.

The MDPI journal *Microorganisms* launched the Special Issue series “Advances in Microbial Cell Factories” [1] in the Microbial Biotechnology Section to capture this rapid momentum. This inaugural volume establishes the foundational framework for the series, focusing heavily on optimized metabolic platforms, overcoming physical bioprocessing limits, and demonstrating rigorous proofs of concept for varied product syntheses. By integrating systems biology with intelligent bioprocess automation, this volume illustrates the immense potential of engineered microbes for sustainability-based disruption of the energy, chemical, and pharmaceutical sectors.

The transition from traditional industrial processes to sustainable biological ones relies on a few critical, cross-cutting pillars that are explored throughout this Special Issue:

- **Metabolic Engineering and Resource Allocation:** The strategic redirection of intracellular carbon fluxes and the optimization of cellular resources (such as minimizing the native proteome) to maximize product titers without impacting cell viability.
- **Predictive Bioprocess Modeling:** The deployment of mathematical, phenomenological, and black-box models to accurately predict fermentation dynamics, scale-up behavior, and shifting microenvironments (such as pH or dissolved oxygen).
- **Bioprocess Automation and Laboratory Evolution:** The use of parallelized, automated bioreactor systems to bypass traditional manual bottlenecks, vastly accelerating adaptive laboratory evolution (ALE) and process optimization.

As the launchpad for a multi-volume series, the 1st Edition has been curated to reflect the most pressing foundational challenges within the biotechnology sector. Rather than merely cataloging new strains, this volume emphasizes the integration of genetic design with reactor-level execution. The manuscripts collected here tackle fundamental bottlenecks: how to coerce a cell to yield more high-value DNA, how to model and control the chaotic shifts of complex solventogenic fermentations, and how to rapidly evolve organisms using next-generation automation. Together, these elements form the critical “Design–Build–Test–Learn” (DBTL) framework necessary for pushing lab-scale curiosities toward industrial-scale realities.

As we reflect on this inaugural collection, it is crucial to highlight the manuscripts that have greatly contributed to the scientific community. The following three featured articles perfectly encapsulate the mission of Volume 1, tackling strain optimization, mathematical process modeling, and hardware automation.

In the manuscript by de la Cruz et al. [2], “*Pentose Phosphate Pathway Flux to Improve Plasmid DNA Production in Engineered E. coli*”, the authors cover a timely topic, demonstrating how to maximize cellular resources for the production of nucleotide therapeutics. The design of optimal microbial cell factories heavily depends on how efficiently a cell allocates its internal resources, so in this highly impactful study, the authors tackle the metabolic burden associated with recombinant plasmid DNA (pDNA) production, a critical component of modern gene therapies and vaccine manufacturing. By utilizing a proteome-reduced strain of *Escherichia coli*, which successfully released 0.5% of its native proteome after just three transcription factors were deleted, the researchers drastically altered intracellular resource management. When cultured in fed-batch mode, this engineered, proteome-reduced strain produced pDNA titers four times greater than its wild-type counterpart. This research stands as a premier example of how stripping away nonessential genetic baggage allows for highly targeted redirection of metabolic fluxes, ultimately establishing a vastly superior platform for high-value biopharmaceutical manufacturing.

Acetone–Butanol–Ethanol (ABE) fermentation by *Clostridium acetobutylicum* is a notoriously complex process, characterized by a distinct biphasic metabolic network, which encompasses an initial acidogenic phase followed by a solventogenic phase. In the manuscript by Kumar et al., “*Exploring the Influence of pH on the Dynamics of Acetone–Butanol–Ethanol Fermentation*” [3], the authors tackle one of the most critical variables dictating the efficiency of this shift: the extracellular pH of the medium. In this featured study, the researchers bridge the information gap between the microscopic cellular system and the macroscopic reactor by developing a robust phenomenological model. Meticulously describing the relationship between biomass growth, desired metabolite synthesis, and dynamic pH profiles, the proposed mathematical model successfully captures the complexities of ABE fermentation. Validated against experimental data, this predictive tool allows bioprocess engineers to virtually simulate and optimize reactor conditions, minimizing the need for costly trial-and-error experimentation while maximizing targeted biofuel yields.

Adaptive laboratory evolution (ALE) is a cornerstone of modern strain development, but traditional serial passaging in manual shake flasks is laborious, slow, and prone to experimental design flaws. In their article, “*Accelerated Adaptive Laboratory Evolution by Automated Repeated Batch Processes in Parallelized Bioreactors*” [4], Bromig and Weuster-Botz engineer a novel, automated alternative by translating the ALE process into a parallelized, liter-scale stirred-tank bioreactor system. Crucially, they integrate off-gas analysis to function as a soft sensor, utilizing a first-principles black-box model to continuously estimate biomass concentration and growth rates in real time. This highly controlled, automated repeated-batch approach eliminates the physical and metabolic inconsistencies of shake flasks. As a result, the authors achieve stable growth rates of *E. coli* on a non-native carbon source (glycerol) that are an astonishing 9.4 times faster than traditional manual methods. This work radically redefines the speed and reliability with which we can evolve microbial cell factories for industrial substrates.

Despite the exceptional progress demonstrated in this 1st Edition, bridging the gap from proof-of-concept to global deployment remains a formidable challenge. While laboratory-scale genetic modifications and automated bench-top reactors frequently yield extraordinary results, translating these metrics to massive industrial production introduces nonlinear biological and physical constraints. Microorganisms in large-scale stainless-steel bioreactors must survive severe fluid-mechanical shear stress, fluctuating nutrient gradients, and transient oxygen deprivation. To mitigate these spatial heterogeneities, the insights from this volume underscore the need for continued investment in bioprocess intensification and scale-down modeling. Future research must build upon the predictive models and automated systems presented here. By proactively “stress-testing” engineered strains within highly monitored, simulated macro-environments, bioprocess engineers can ensure metabolic stability is maintained before investing heavily in physical scale-up infrastructure. The advances showcased in this volume confidently light the path forward, demonstrating that the fusion of synthetic biology, computational modeling, and automated bioprocessing is the key to unlocking the circular bioeconomy.
